# Transcriptome Profiling of *Gossypium anomalum* Seedlings Reveals Key Regulators and Metabolic Pathways in Response to Drought Stress

**DOI:** 10.3390/plants12020312

**Published:** 2023-01-09

**Authors:** Wei Ji, Huan Yu, Yixin Shangguan, Jing Cao, Xianglong Chen, Liang Zhao, Qi Guo, Peng Xu, Xinlian Shen, Zhenzhen Xu

**Affiliations:** Key Laboratory of Cotton and Rapeseed (Nanjing), Ministry of Agriculture and Rural Affairs, The Institute of Industrial Crops, Jiangsu Academy of Agricultural Sciences, Nanjing 210014, China

**Keywords:** *Gossypium anomalum*, drought stress, transcriptome, molecular mechanism, pathway

## Abstract

Drought stress is a key limiting factor for cotton (*Gossypium* spp.) growth, production, development, and production worldwide. Some wild diploid cotton species are remarkably tolerant of water deficit and constitute an important reservoir for understanding the molecular mechanisms of *Gossypium* spp. drought tolerance and improving cultivated upland cotton. Here, we utilized RNA-Seq technology to characterize the leaf transcriptomes of a wild African diploid cotton species, *Gossypium anomalum*, under drought stress. A total of 12,322 differentially expressed genes (DEGs) were identified after mapping valid clean reads to the reference genome of *G. anomalum*, of which 1243 were commonly differentially expressed at all stages of drought stress. These genes were significantly enriched for molecular functions Gene Ontology terms related to cytoskeleton, hydrolase activity, cellular redox, and binding. Additionally, a substantial proportion of enriched biological process terms concerned cell or subcellular processes, while most in the cellular components category concerned membrane function and photosynthesis. An enrichment analysis against the Kyoto Encyclopedia of Genes and Genomes showed the top significantly enriched pathways to be photosynthesis-antenna proteins, amino sugar and nucleotide sugar metabolism, starch and sucrose metabolism, MAPK signaling pathway, glutathione metabolism, and plant hormone signal transduction. The DEGs also exhibited interestingly significant enrichments for drought stress-induced tandemly repeated genes involved in iron ion binding, oxidoreductase activity, heme binding, and other biological processes. A large number of genes encoding transcription factors, such as MYB, bHLH, ERF, NAC, WRKY, and bZIP, were identified as playing key roles in acclimatizing to drought stress. These results will provide deeper insights into the molecular mechanisms of drought stress adaptation in *Gossypium* spp.

## 1. Introduction

Drought is one of the major abiotic stresses that limits plant growth, development, and production worldwide [1,2]. Some plants have the ability to maintain physiological activities under drought conditions through the modulation of key regulators or metabolic pathways that could reduce or repair drought-induced damage [3,4]. Several main strategies can be distinguished: ion and osmotic homeostasis that maintain cell morphology or lower the osmotic potential, stress damage control and repair, and growth regulation [5]. Consequently, understanding the molecular responses of plants to drought stress so as to improve varieties with a high yield potential and enable them to adapt to this adverse environmental condition has been a point of focus for molecular breeders [6,7,8,9,10,11,12]. A transcriptomic analysis has been applied to detect dynamic changes in the expression of regulatory or functional genes and has provided in-depth knowledge of the biological processes and key pathways that underlie plant adaptation to drought stress [13,14,15,16].

Cotton (*Gossypium* spp.) is cultivated worldwide and constitutes the preeminent source of natural fibers for the textile industry [17]. The domesticated allotetraploid species *Gossypium hirsutum* L. (2n = 4x = 52, (AD)_1_), commonly known as upland cotton, is responsible for ~90% of the world’s annual cotton production on account of its relatively high yield potential and moderate fiber quality [18]. However, extreme drought affects its growth, productivity, and fiber quality [19]. In addition, the narrow genetic basis of modern cultivated upland cotton species makes it a challenge for scientists to further improve their drought tolerance. In contrast, most wild diploid cotton species grow in arid and semi-arid areas and frequently experience periodic drought and high temperature extremes [20]. The long-term adaptive evolution of these wild species suggests that they may possess morphological, physiological, and molecular mechanisms for drought tolerance, and hence harbor responsive loci or genes that could be used to improve the drought tolerance of modern cultivated upland cotton.

*G. anomalum* (B_1_B_1_, 2n = 2x = 26), a diploid wild species in *Gossypium* with a close relative A-genome species, grows widely in arid to extremely arid parts of Africa across the southern Sahara from the Sudan to the upper reaches of the valley of the Baraka River in Eritrea [21]; thus, the plants are frequently exposed to harsh environmental conditions. This species offers a wealth of breeding potential for desirable traits, such as good fiber quality, immunity to certain bacterial diseases, male sterility through cytoplasm, resistance to insect pests, and tolerance to water deficit [22,23]. Morphologically, *G. anomalum* exhibits phenotypes that make plants more resistant to drought stress, such as small and deeply parted palmate leaves, thick and hard seed coats, and dense hairs. Previous literature has documented its drought tolerance [22,24,25,26]. However, detailed study of the molecular mechanisms underlying drought tolerance in *G. anomalum* has been limited. To date, there were no transcriptomic data of *G. anomalum* under drought stress deposited in the NCBI database (https://www.ncbi.nlm.nih.gov/, accessed on 14 October 2022).

Here, we performed RNA-Seq with the aim of characterizing dynamic changes of the molecular responses to drought stress in *G. anomalum* seedlings during different stages of stress and to identify key regulators or pathways involved in this response. These transcriptomic data provide a useful resource for further research that will ultimately construct a comprehensive understanding of the molecular mechanisms for drought tolerance in *Gossypium* spp. that might be of value in cotton breeding programs.

## 2. Results

### 2.1. Physiological and Biochemical Evaluation of G. anomalum Seedlings in Response to Drought Treatment

**Figure 1:**
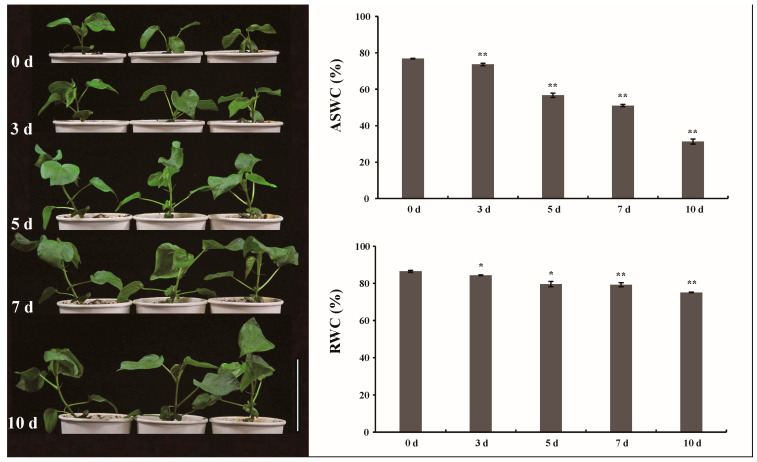
Phenotype of drought-stressed *G. anomalum* seedlings, the RWC of *G. anomalum* seedlings and ASWC. RWC: the relative water content of *G. anomalum* seedlings, ASWC: absolute soil water content. * *p* < 0.05, ** *p* < 0.01, Student’s *t* test.

The relative water content (RWC) of *G. anomalum* seedlings and absolute soil water content (ASWC) decreased especially in later periods (7 d and 10 d) under drought stress (Figure 1). This was in agreement with the appearance of visual symptoms of drought stress in *G. anomalum* seedlings. Compared with the control (0 d), plants at 7 d and 10 d exhibited a slight leaf curl and chlorosis (Figure 1).

**Figure 2:**
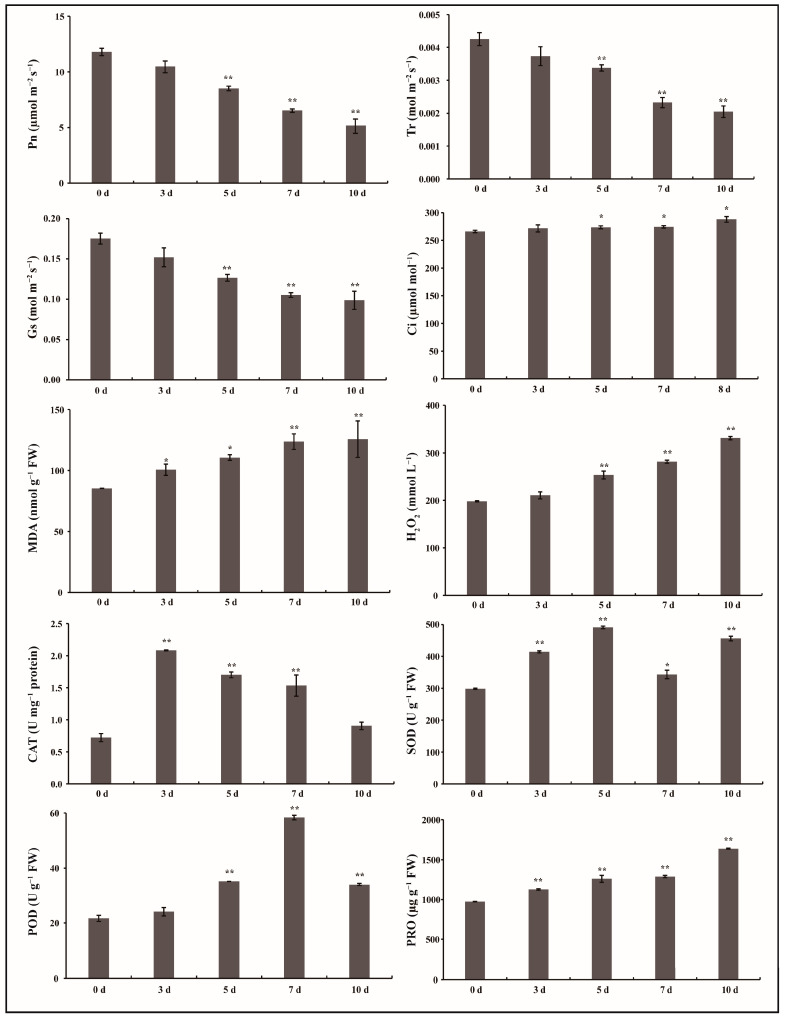
Physiological and biochemical evaluation of *G. anomalum* seedlings in response to drought treatment. Pn: net photosynthetic rate, Tr: transpiration rate, Gs: stomatal conductance, Ci: intercellular CO_2_ concentration, MDA: malondialdehyde, H_2_O_2_: hydrogen peroxide, CAT: catalase, SOD: superoxide dismutase, POD: peroxidase, PRO: proline. * *p* < 0.05, ** *p* < 0.01, Student’s *t* test.

Photosynthetic parameters, including the net photosynthetic rate (Pn, μmol m^−2^ s^−1^), transpiration rate (Tr, mol m^−2^ s^−1^), and stomatal conductance (Gs, mol m^−2^ s^−1^), also significantly decreased (*p* < 0.01) at 5 d, 7 d, and 10 d under drought stress, while the intercellular CO_2_ concentration (Ci, µmol mol^−1^) steadily increased (*p* < 0.05) (Figure 2), indicating that photosynthetic functions were inhibited under drought stress, especially at later periods.

Conversely, with drought stress treatment, the relative content of malondialdehyde (MDA) significantly increased (*p* < 0.05, *p* < 0.01) in *G. anomalum* leaves (Figure 2). Similarly, hydrogen peroxide (H_2_O_2_) content showed no obvious changes at 3 d into treatment but was significantly increased (*p* < 0.01) at 5 d, 7 d, and 10 d (Figure 2). These data demonstrate that *G. anomalum* plants undergo relatively serious oxidative and membrane damage at later stages of drought stress. Plants have complex antioxidative defense systems to maintain reactive oxygen species (ROS) scavenging ability and control intracellular homeostasis [27,28]. The second leaf from the top of five individual seedlings was assessed for catalase (CAT), superoxide dismutase (SOD), and peroxidase (POD) activity, and also proline (PRO) content at 0, 3, 5, 7, and 10 d of drought treatment and was found to significantly increase all values (*p* < 0.05, *p* < 0.01) compared to the control (0 d) (Figure 2). CAT activity reached its highest value of drought stress at 3 d, SOD activity at 5 d, POD activity at 7 d, and PRO at 10 d (Figure 2). These results suggest that the CAT, SOD, POD, and PRO played their respective roles in different phases of drought stress.

### 2.2. Analyses of RNA-Seq Data

To conduct a broader survey of genes involved in drought tolerance, RNA-Seq was performed for *G. anomalum* leaves at 0, 3, 5, 7, and 10 d of drought treatment with three replications. Each sample was represented by at least 39,188,662 raw reads, which is sufficient for a quantitative analysis of gene expression. After quality checks and adapter removal, 39,142,792–50,408,364 clean reads were obtained for further analysis. The Q20 and Q30 values across all samples were ≥ 96.80% and 91.48%, respectively. Of the total clean reads, 91.59–95.79% were mapped and 89.14–93.30% uniquely so to the reference sequence of *G. anomalum* [29]. Additionally, all valid clean short reads were *de novo* assembled, resulting in 1820 new contigs with a mean length of 1383 bp. A summary of RNA-Seq data is given in Table S1.

### 2.3. Differential Gene Expression at Different Stages of Drought Stress

**Figure 3:**
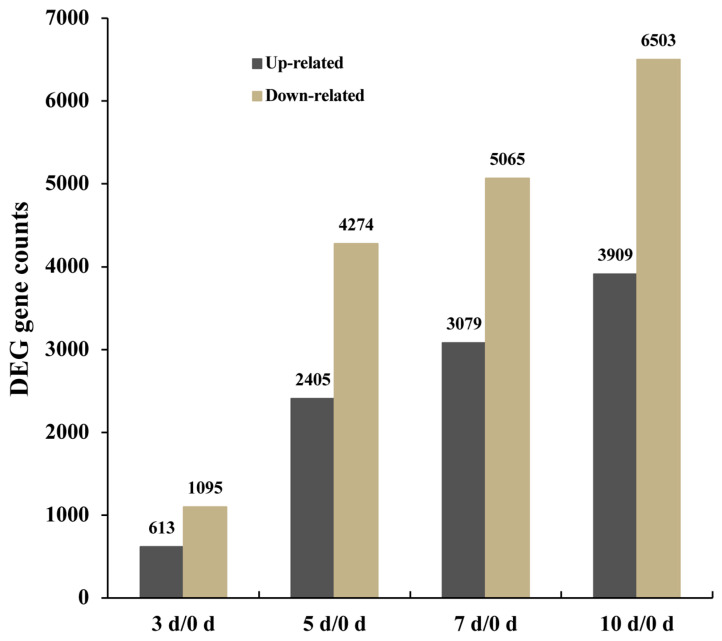
Number of differentially expressed genes in *G. anomalum* under drought stress. 0 d, 3 d, 5 d, 7 d, and 10 d refer to 0, 3, 5, and 10 days after drought stress.

To identify genes that undergo significant expression changes during drought stress in *G. anomalum*, the 3 d, 5 d, 7 d, and 10 d libraries were compared with the control (0 d). This determination of expression changes uncovered 12,322 differentially expressed genes (DEGs) at the threshold of |log_2_FoldChange| ≥ 1 and *p*_adj_ ≤ 0.05, of which 11,606 were mapped on the reference genome of *G. anomalum* and 716 were annotated as novel in the current study (Table S2). Regarding each individual data set, 1708 (613 up-regulated and 1095 down-regulated), 6679 (2405 up-regulated and 4274 down-regulated), 8144 (3079 and 5065 down-regulated), and 10,412 (3909 up-regulated and 6503 down-regulated) DEGs were respectively identified in leaf samples at 3, 5, 7, and 10 d of drought treatment (Figure 3 and Table S2). The number of DEGs sharply increased as the drought proceeded, and the down-regulation of genes predominated throughout the drought period (Figure 3).

To verify the reliability of the obtained drought-responsive gene expression profiles, the relative expression of 12 randomly selected genes was confirmed by quantitative real-time PCR (qRT-PCR) using gene-specific primers and the relative threshold cycle (Ct) method (2^−∆Ct^ method) (Table S3). These selected genes encoded abscisic acid-insensitive 5-like protein (*Goano05G2948*), probable glutathione S-transferase (*Goano03G0271*), WRKY transcription factor 18 (*Goano06G1393*), scarecrow-like protein 13 (*Goano05G1959*), myb-related protein 108 (*Goano11G1258*), myb-related protein Myb4 (*Goano13G2422*), phenylpropanoid: glucosyltransferase 1 (*Goano13G2723*), phosphorylated protein of 32 kDa (*Goano12G1626*), protein phosphatase 2C (PP2C, *Goano00G0175*), catalase isozyme 1 (*Goano01G1791*), ubiquitin carboxyl-terminal hydrolase 12 (*Goano10G2669*), and proline transporter 1 (*Goano10G3130*). The expression patterns of these 12 selected drought-responsive genes, as determined by qRT-PCR, were obviously consistent with the RNA-seq data (Figure S1), thus confirming the reliability of the transcriptome sequencing.

### 2.4. Gene Ontology (GO) Annotation and Enrichment Analysis

**Figure 4:**
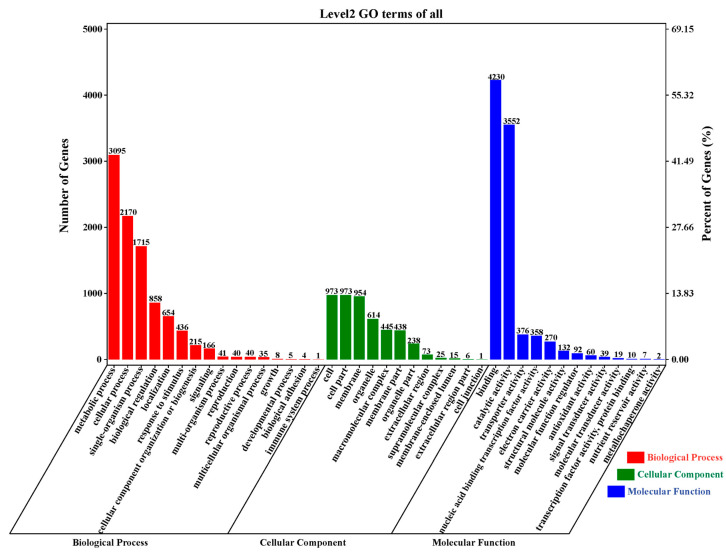
Gene ontology (GO) annotation distribution for differentially expressed genes in *G. anomalum* under drought stress. Histograms represent the functional distribution; the left *y*-axis shows the number of genes, and the right *y*-axis shows a percentage of the number of genes.

To decipher the biological processes affected by drought stress, a GO-based classification of the DEGs was carried out using the biological process (BP), cellular component (CC), and molecular function (MF) terms. A total of 7234 DEGs were annotated with 42 functional terms (Figure 4 and Table S4); however, a considerable proportion (41.29%) of DEGs could not be mapped to any GO category. The major subcategories under BP were metabolic process, cellular process, and single organism process, while the largest proportion in the CC category consisted of the cell membrane with the cell part and membrane (Figure 4). Finally, in the MF category, a significant proportion of DEGs were as annotated with binding, catalytic activity, transporter activity, and nucleic acid binding transcription factor activity (Figure 4).

**Figure 5:**
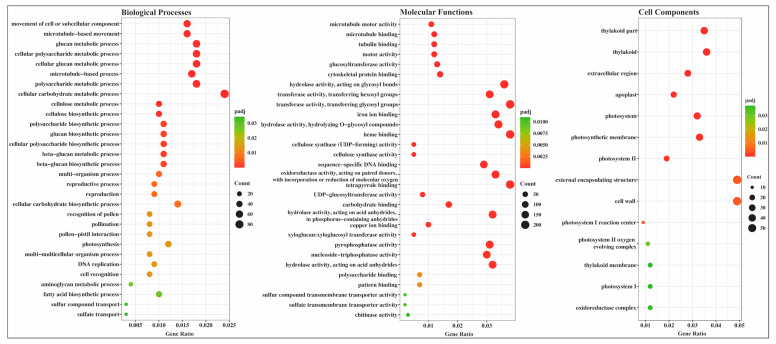
Gene ontology (GO) enrichment for differentially expressed genes in *G. anomalum* under drought stress. The closer to red, the more the differential genes contained under each function are represented by the size of the dots. Only top 30 significantly enriched GO terms of each category are displayed in the DEGs.

The evaluation of significant enrichments among DEGs revealed 90 GO terms as enriched (*p*_adj_ ≤ 0.05), including 43 MF terms, 31 BP terms, and 16 CC terms (Figure 5 and Table S5). The majority of enriched MF terms related to the cytoskeleton (e.g., GO:0003777: microtubule motor activity; GO:0008017: microtubule binding; GO:0015631: tubulin binding; GO:0003774: motor activity; GO:0008092: cytoskeletal protein binding), hydrolase activity (e.g., GO:0016798: hydrolase activity, acting on glycosyl bonds; GO:0004553: hydrolase activity, hydrolyzing O-glycosyl compounds; GO:0016818: hydrolase activity, acting on acid anhydrides, in phosphorus-containing anhydrides; GO:0016817: hydrolase activity, acting on acid anhydrides; GO:0016788: hydrolase activity, acting on ester bonds; GO:0016799: hydrolase activity, hydrolyzing N-glycosyl compounds), cellular redox (e.g., GO:0016705: oxidoreductase activity, acting on paired donors, with incorporation or reduction of molecular oxygen; GO:0016620: oxidoreductase activity, acting on the aldehyde or oxo group of donors, NAD or NADP as acceptor), and binding (e.g., GO:0043565: sequence-specific DNA binding; GO:0005507: copper ion binding; GO:0005506: iron ion binding; GO:0046906: tetrapyrrole binding; GO:0020037: heme binding) (Figure 5 and Table S5). In the BP category, a significant proportion of the clusters concerned the cell or subcellular processes (e.g., GO:0006928: movement of cell or subcellular component; GO:0007018: microtubule-based movement; GO:0007017: microtubule-based process; GO:0006073: cellular glucan metabolic process; GO:0044264: cellular polysaccharide metabolic process; GO:0044262: cellular carbohydrate metabolic process; GO:0008037: cell recognition; GO:0044706: multi-multicellular organism process; GO:0033692: cellular polysaccharide biosynthetic process; GO:0034637: cellular carbohydrate biosynthetic process) (Figure 5 and Table S5). Among the CC group, most enriched terms were concerned with membrane function (e.g., GO:0009579: thylakoid; GO:0044436: thylakoid part; GO:0005576: extracellular region; GO:0005618: cell wall; GO:0030312: external encapsulating structure; GO:0042651: thylakoid membrane; GO:0019898: extrinsic component of membrane; GO:0071944: cell periphery) and photosynthesis (e.g., GO:0009521: photosystem; GO:0034357: photosynthetic membrane; GO:0009523: photosystem II; GO:0009538: photosystem I reaction center; GO:0009654: photosystem II oxygen evolving complex; GO:0009522: photosystem I) (Figure 5 and Table S5).

### 2.5. Kyoto Encyclopedia of Genes and Genomes (KEGG) Annotation and Enrichment Analysis

**Figure 6:**
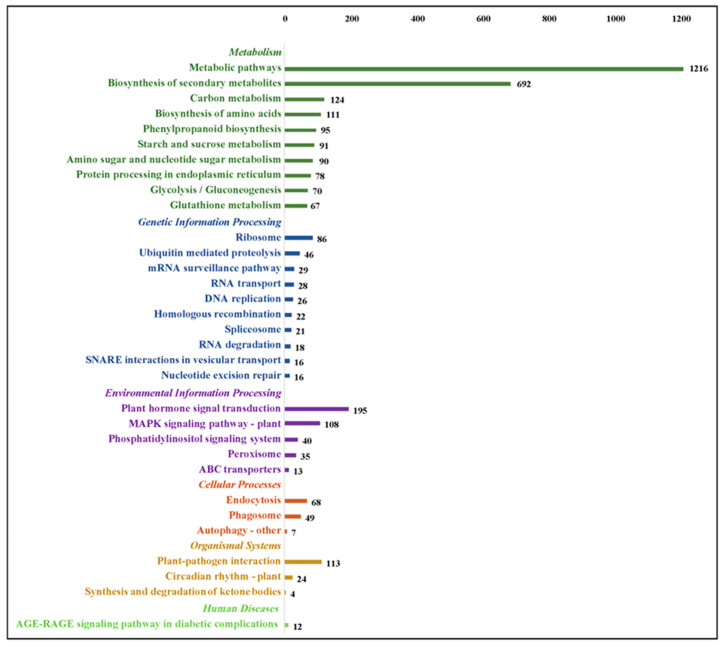
Kyoto Encyclopedia of Genes and Genomes (KEGG) annotation distribution for differentially expressed genes in *G. anomalum* under drought stress. Only top 10 pathway terms are displayed in each group.

To get a broader view of the molecular mechanisms underlying drought stress in *G. anomalum*, the functional annotation of 12,322 DEGs was performed by searching the KEGG database. In total, 2200 DEGs were found to be associated with six definite pathway groups, including 4472 in metabolism, 391 in environmental information processing, 379 in genetic information processing, 124 in cellular processes, 141 in organismal systems, and 12 in human diseases (Figure 6 and Table S6). Since one gene may be involved in multiple pathways, the total count of pathway annotations was greater than the number of individual DEGs with functional annotation in KEGG. Of DEGs in the metabolism cluster, the pathway breakdown was as follows: 1216 in metabolic pathways (ko01100), 692 in the biosynthesis of secondary metabolites (ko01110), 124 in carbon metabolism (ko01200), 111 in the biosynthesis of amino acids (ko01230), 95 in phenylpropanoid biosynthesis (ko00940), and 91 in starch and sucrose metabolism (ko00500) (Figure 6). For the environmental information processing group, the major pathways were the plant hormone signal transduction (ko04075) and MAPK signaling pathway (ko04016), which included 195 and 108 DEGs, respectively (Figure 6). With regard to genetic information processing, cellar processes, and organismal systems, the most represented pathways were the ribosome (86 DEGs), endocytosis (68 DEGs), and plant-pathogen interaction (113 DEGs), respectively (Figure 6).

**Figure 7:**
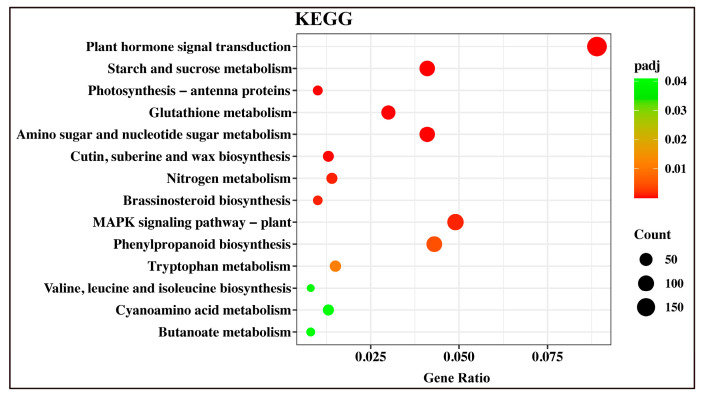
Scatter plot of KEGG pathway enrichment statistics. Rich factor is the ratio of differentially expressed gene numbers annotated in this pathway term. Greater rich factor means more demanding.

We also constructed a scatter plot to identify the significant enrichment of KEGG functions. This analysis represented the number of DEGs annotated to the function divided by the number of genes annotated to the function. This analysis yielded 27 important KEGG pathways that were significantly enriched among the DEGs, with the top pathways being photosynthesis-antenna proteins (ko00196), amino sugar and nucleotide sugar metabolism (ko00520), starch and sucrose metabolism (ko00500), MAPK signaling pathway (ko04016), glutathione metabolism (ko00480), and plant hormone signal transduction (ko04075) (Figure 7 and Table S7).

### 2.6. Functional Annotation of Common DEGs

**Figure 8:**
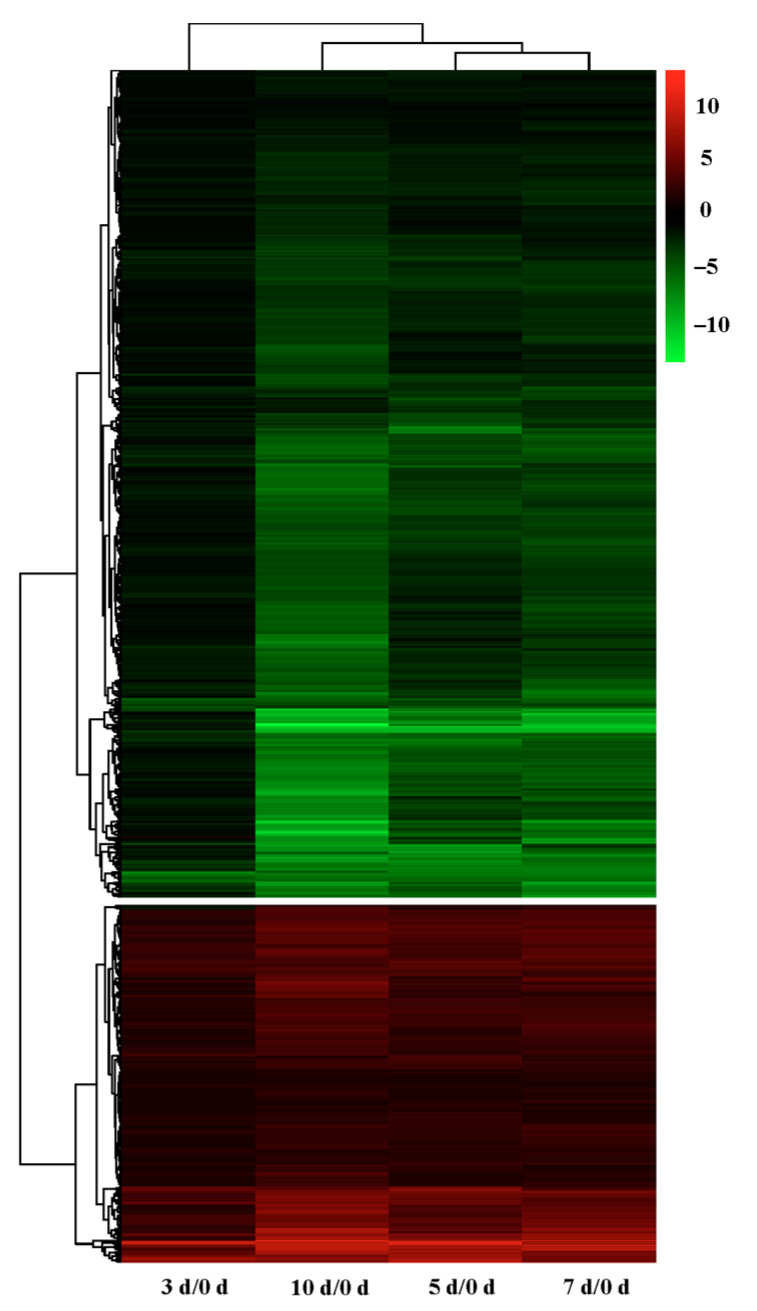
Hierarchical cluster analysis of 1243 common differentially expressed genes in *G. anomalum* under drought stress. The up-regulated genes are marked in red, while down-regulated genes are marked in green. The DEGs of 3 d-treated sample are divided in a separate group, while 5 d samples are grouped closely with those in at 7 d and 10 d.

A total of 1243 genes exhibited differential expression in all stages of drought stress (Figure 8). The expression patterns of these genes in response to drought stress were clustered by a hierarchical algorithm, which reveals 3 d samples to segregate separately, representing the early stage of drought stress (Figure 8). Meanwhile, 5 d samples grouped closely with those at 7 d and 10 d, which together represented the late stage of drought stress (Figure 8).

**Figure 9:**
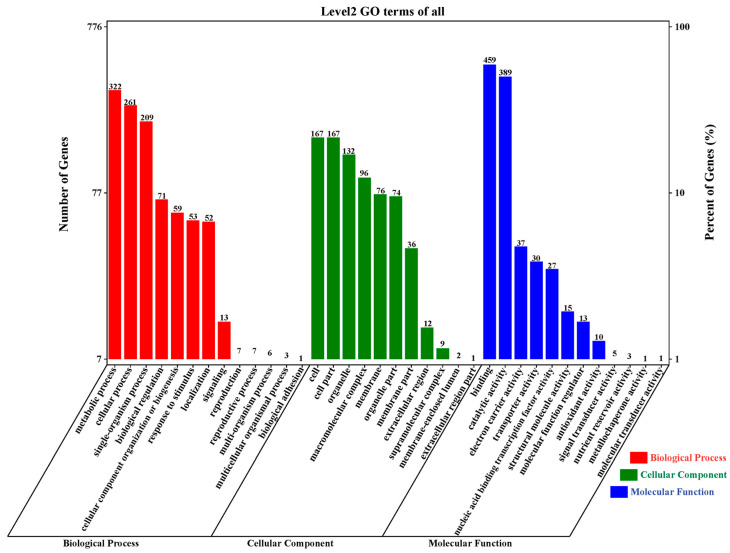
GO function analysis of common differentially expressed genes in *G. anomalum* under drought stress. Histograms represent the functional distribution; the left *y*-axis shows the number of genes, and the right *y*-axis shows a percentage of the number of genes.

Of these 1243 common DEGs, 776 genes were found to have GO annotations based on sequence similarity. These genes represented 36 terms in all, which included 13 BP, 11 CC, and 12 MF terms. The most-represented GO terms were metabolic, cellular, and single-organism process in the BP category; cell part, cell, and organelle in the CC category; and binding and catalytic activity in the MF category. The GO terms of these 1243 common DEGs are presented in Figure 9.

**Figure 10:**
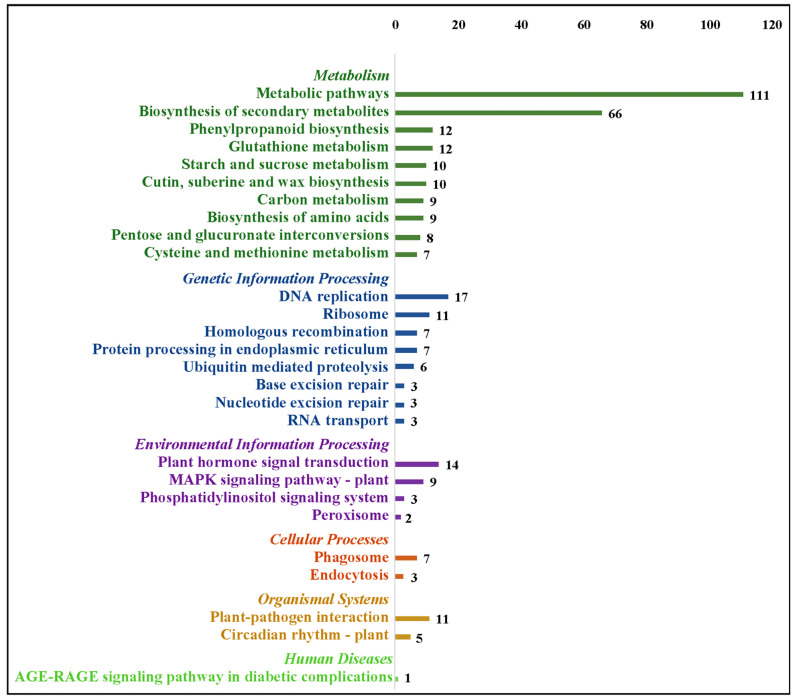
KEGG function analysis of common differentially expressed genes. Only top 10 pathway terms are displayed in each group.

The annotation of these 1243 common DEGs based on the KEGG database revealed 225 genes to be associated with six definite pathway groups, including 396 in metabolism, 58 in genetic information processing, 29 in environmental information processing, ten in cellular processes, 16 in organismal systems, and one in human diseases (Figure 10 and Table S9). A large proportion of the annotated genes belonged to the pathways of the metabolic pathway and biosynthesis of secondary metabolites in metabolism, DNA replication and ribosome in genetic information processing, plant hormone signal transduction and the MAPK signaling pathway in environmental information processing, phagosome in cellular processes, and plant-pathogen interaction in organismal systems (Figure 10).

### 2.7. Identification of Drought-Responsive Tandemly Repeated Genes and Transcriptional Factors (TFs)

**Figure 11:**
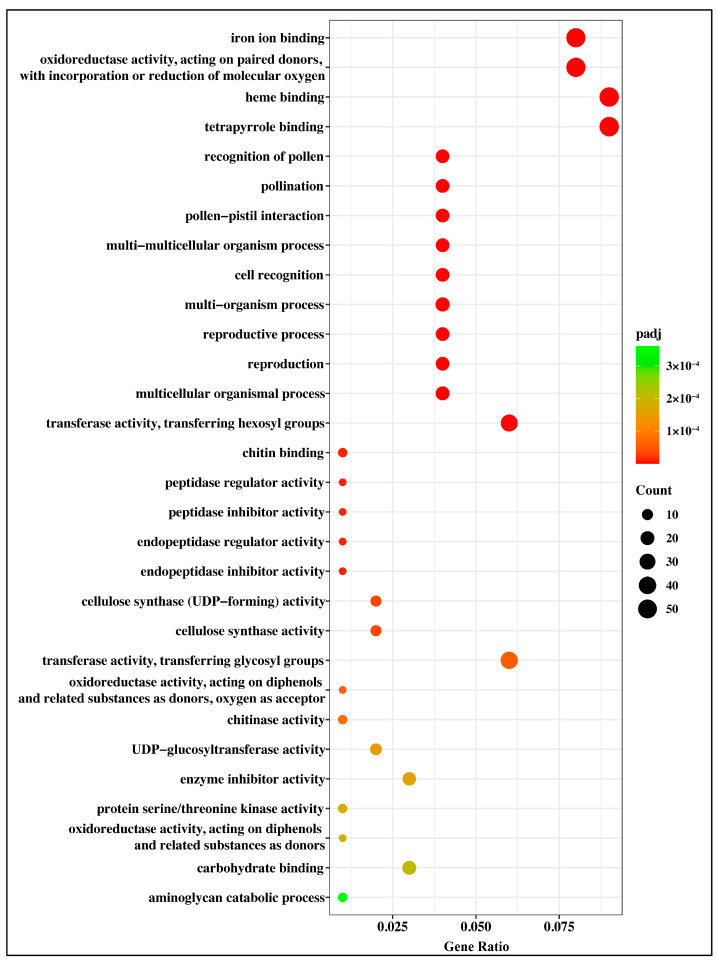
Gene ontology (GO) enrichment for drought-responsive tandemly repeated genes in *G. anomalum*. The closer to red, the more the differential genes contained under each function are represented by the size of the dots.

A total of 5305 tandemly repeated genes were identified in the *G. anomalum* genome, of which 704 were differentially expressed in response to drought stress. A GO enrichment analysis indicated these drought stress-modulated tandemly repeated genes to be associated with 82 GO terms, of which the top four most significantly enriched were GO:0005506 (iron ion binding), GO:0016705 (oxidoreductase activity, acting on paired donors, with incorporation or reduction of molecular oxygen), GO:0020037 (heme binding), and GO:0046906 (tetrapyrrole binding) (Figure 11 and Table S10).

**Figure 12:**
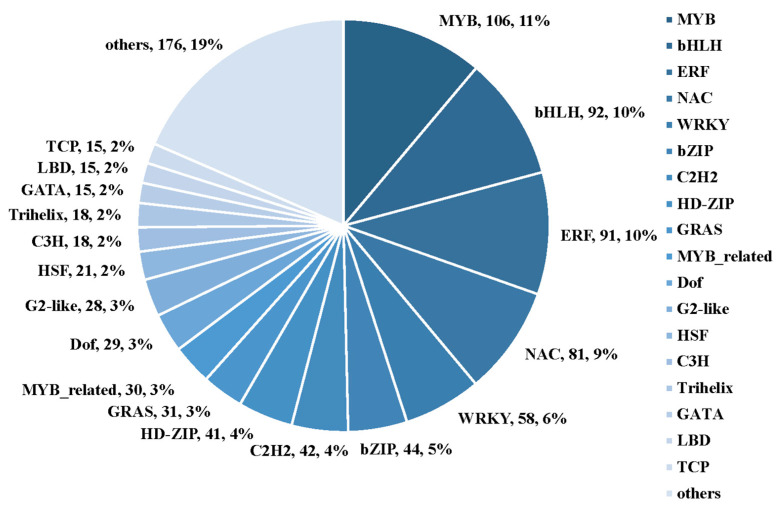
The distribution of TFs genes families. The size of the sector represents the frequency of each TFs. Top five TFs are the MYB, bHLH, ERF, NAC, and WRKY family, which might be involved in response to the drought stress.

Pairwise comparisons of each drought treatment timepoint against the control (i.e., 3 d vs. 0 d, 5 d vs. 0 d, 7 d vs. 0 d, and 10 d vs. 0 d) revealed a total of 951 transcription factors (TFs) to be differentially expressed (Figure 12 and Supplementary Table S11). These TFs represented 48 different families. In particular, we observed that TFs belonging to the MYB family were predominantly altered under drought stress, followed by members of the bHLH, ERF, NAC, WRKY, bZIP, C2H2, HD-ZIP, and GRAS families.

## 3. Discussion

The diversity of modern cultivated upland cotton has been dramatically constricted due to extensive selection within limited resources, and it has become a major bottleneck in improving cotton drought tolerance. Wild cotton relatives represent potentially valuable gene pools and are the primary source of drought-responsive genes. Here, we performed an RNA-Seq analysis of leaves from the wild African cotton species *G. anomalum* during drought stress and identified 12,322 DEGs that are altered in response to drought stress. These DEGs exhibited significant enrichments for molecular functions GO terms related to cytoskeleton, hydrolase activity, cellular redox, and binding; biological processes terms involving the cell or subcellular processes; and cellular component terms concerned with membrane function and photosynthesis. These findings are in agreement with other studies [30,31,32,33,34,35] and suggest that the genes associated with these GO terms might be involved in drought tolerance. Notably, DEGs relating to photosynthesis, including genes involved in the photosystem, photosynthetic membrane, photosystem II, photosynthesis, photosystem I reaction center, photosystem II oxygen evolving complex, and photosystem I, were typically down-regulated under drought stress, indicating that photosynthetic functions were inhibited (especially from 5 d on). These results are consistent with observed photosynthetic parameters, in that Pn, Tr, and Gs were significantly decreased at 5 d, 7 d, and 10 d of drought treatment, while Ci steadily increased. A KEGG enrichment analysis similarly showed that top significantly enriched pathways among DEGs concerned photosynthesis-antenna proteins, amino sugar and nucleotide sugar metabolism, starch and sucrose metabolism, MAPK signaling pathway, glutathione metabolism, and plant hormone signal transduction. As described in a previous study [31], drought stress induces the MAPK signaling pathway and signal transduction through plant hormones, which have strong effects on biosynthesis and metabolism and might led to a decline in photosynthesis.

Broadly speaking, plant hormones regulate stress-mediated signaling pathways in plant adaptation to abiotic stress [36]. Concerning drought stress in particular, the abscisic acid (ABA) signaling pathway is known to play a central role and regulate the transcription of MAPK genes [17]. Previous studies have illustrated that drought significantly increases the expression of ABA-mediated genes in cotton [37]. The overexpression of the ABA-induced cotton genes *GhCBF3*, *AREB1*, and *AREB2* in cotton has been shown to improve drought tolerance due to the higher chlorophyll, proline, and relative water contents [38,39]. Additionally, drought tolerance has been positively correlated with the activation of the ABA receptor gene *GhPYL9-11A* in transgenic *Arabidopsis* plants [40]. This study identified 80 genes that are positively regulated during drought stress in *G. anomalum*. These included two genes encoding SnRK2 (*Goano08G2297* and *Goano06G0436*), which improves drought tolerance by enhancing ABA signaling [41], and two encoding PYR/PYL (*Goano08G1795* and *Goano04G2489*), an ABA receptor that takes part in triggering ABA responses [42]. Several MAPK-related genes, such as *GhMPK3*, *GhMAP3K1*, *GhMKK4*, and *GhMPK6*, have previously been implicated in the responses of *G. hirsutum* L. and *G. barbadense* L. to drought stress [43,44,45,46,47,48,49,50,51]. *GhMAP3K49* is significantly induced by ABA and ROS, and its product interacts with GhMKK9 and GhMKK4 in a complete cascade [52]. A novel GhMAP3K15-GhMKK4-GhMPK6-GhWRKY59 phosphorylation loop has also been reported that regulates the GhDREB2-mediated and ABA-independent drought response in cotton [53]. In this study, 54 MAPK-related genes, such as *MKK2* (*Goano07G0148* and *novel.1799*), *MPK6* (*Goano01G1166*), *MARK3/6* (*Goano01G1166* and *Goano02G1761*), *MKK9* (*Goano12G0072*), and *MAPK17/18* (*Goano10G0114*) were up-regulated, especially in the later periods stage of drought stress, which heavily implicates MAPK pathways in the response to drought. To date, only one gene conferring drought tolerance, namely *Goano05G0268*, has been cloned from *G. anomalum* [29]. Further analysis of these drought-responsive genes will assist in laying a foundation for molecular manipulation towards the development of new upload cotton cultivars with improved drought tolerance.

The high proportions of repetitive elements and transposons in the genome makes it easy to generate duplicated gene fragments/alleles with no function, neo-function, redundant function, or enhanced function [54,55]. For example, in wheat, the constitutive expression of *TaCYP81D5*, a gene isolated from a salt stress-related hotspot region which consisted of five tandemly distributed copies, enhanced the salinity tolerance at both seedling and reproductive stages via accelerating ROS [56]. The knockout of *TaCYP81D5* alone had no effect on salinity tolerance, but the knockdown of all *TaCYP81D* members in the cluster induced the sensitivity to salt stress [56]. In this work, *G. anomalum* exhibited an interestingly significant enrichment for drought stress-induced tandemly repeated genes involved in iron ion binding, oxidoreductase activity, heme binding, tetrapyrrole binding, transferase activity, and other biological processes. These enrichments suggest a characteristically higher overall stress-tolerant evolution of the species.

The common DEGs that exhibited an altered expression throughout the drought treatment in this study included a large proportion (467/1243, 41.29%) without a GO annotation. Moreover, many of these genes exhibited a substantial change in expression levels during drought stress. These results suggest that more unknown genes or complex pathways are involved in the response to drought tolerance in *Gossypium* spp. Further analysis of these drought-responsive genes without functional annotation will enhance our understanding of drought tolerance mechanisms in plants.

## 4. Materials and Methods

### 4.1. Preparation of Plant Material

Seeds of the wild *Gossypium* species *G. anomalum* were pre-germinated in distilled deionized water in a plant incubator at 60% humidity, 16 h day/8 h night, and a day/night temperature of 28/23 °C. Then, germinated seeds were planted in commercial sterilized soil and cultured in the same conditions for 15 days.

The uniformly developed seedlings with three true leaves were first well-watered, and then water was withheld to induce drought stress. The second leaf from the top of five individual seedlings in each biological replicate was collected at 0, 3, 5, 7, and 10 d after beginning the drought treatment. All samples were frozen immediately in liquid nitrogen and then stored at −70 °C for the extraction of RNA.

### 4.2. Determination of Physiological and Biochemical Indexes

The relative water content (RWC) of *G. anomalum* and absolute soil water content (ASWC) were determined by the drying method [57,58].

The LI-6800 portable photosynthesis system (LI-COR Inc., Lincoln, NE, USA) was used to observe the photosynthetic parameters of the seedlings. The CO_2_ concentration in the reference leaf chamber was controlled at a constant value of 400 μmol mol^−1^, and the photosynthetically active radiation and air relative humidity were set to 1500 μmol m^−2^ s^−1^ and 50%, respectively. The experimental apparatus automatically recorded the Pn, Tr, Gs, and Ci of the second leaf from the top of five individual seedlings at 0, 3, 5, 7, and 10 d after drought stress.

The MDA, H_2_O_2_, and PRO contents, CAT, SOD, and POD activity of the second leaf from the top of five individual seedlings at 0, 3, 5, 7, and 10 d after drought stress were measured by assay kits (JianCheng, Nanjing, China).

### 4.3. Library Preparation for Transcriptome Sequencing

Total RNA for each sample was extracted using the RNAprep Pure Plant Plus Kit (TIANGEN, Beijing, China) following the manufacturer’s instructions. The total RNA amounts and integrity were assessed using the RNA Nano 6000 Assay Kit of the Bioanalyzer 2100 system (Agilent Technologies, Santa Clara, CA, USA).

RNA-seq libraries were constructed using the Illumina TruSeq Stranded RNA Library Preparation Kit (Illumina, San Diego, CA, USA), and then quantified with a Qubit2.0 Fluorometer (Invitrogen, Carlsbad, CA, USA). After all libraries were constructed, they were pooled according to the effective concentration and the target amount of data, then sequenced on the Illumina NovaSeq 6000 platform (150 bp paired-end).

### 4.4. Differentially Expressed Genes (DEGs) Analysis

After the adapter removal and quality checks, we aligned the valid clean data to the reference genome of *G. anomalum* [29] using the Hisat2 (v2.0.5) software [59]. To supplement the reference genome, the same data were also *de novo* assembled using StringTie (v1.3.3b) with a novel network flow algorithm [60]. Subsequently, featureCounts v1.5.0-p3 [61] was used to estimate gene expression levels, determined in fragments per kilobase of exon model per Million mapped fragments (FPKM) based on the gene length and read count.

Using the *DESeq2* R package (1.20.0) [62], a differential expression analysis was performed by comparing the 3 d, 5 d, 7 d, and 10 d libraries with the control (0 d). After controlling for the false discovery rate using the approach of Benjamini and Hochberg, differentially expressed genes (DEGs) were identified as those having |log_2_FoldChange| ≥ 1 and *p*_adj_ ≤ 0.05.

### 4.5. GO and KEGG Analysis

The functional annotation of all genes was performed according to the best BLAST hit by BLASTP (E-value ≤ 1 × 10^−5^ against the Gene Ontology (GO) and Kyoto Encyclopedia of Genes and Genomes (KEGG) databases. Upon the GO functional classification and KEGG pathway analysis, the functional category distribution frequency was respectively calculated for biological process level 2 terms and metabolic pathways. Compared to the reference gene background of *G. anomalum*, a GO and KEGG enrichment analysis was performed for DEGs in each the four comparisons using an FDR adjusted *p*-value ≤0.05 as the cutoff. The number of DEGs in every GO term and KEGG pathway were displayed using the *edgeR* package.

### 4.6. Tandemly Repeated Genes and Transcription Factors (TFs) Annotation

To identify putative transcription factors among the DEGs, the consensus sequences were compared with protein sequences present in the Plant Transcription Factor database (PlnTFDB) using BLASTx (version 5.0).

To identify tandemly repeated genes, all genes anchored to the chromosomes were compared by BLASTP (e-value < 1 × 10^−20^, and homologous genes with a maximum of five intervening genes were defined as tandem duplicates [63]. A KEGG enrichment analysis of these tandemly repeated genes was performed using the *clusterProfiler* package with the cutoff set at the adjusted *p* value < 0.05 [64].

### 4.7. Data Validation by qRT-PCR

For qRT-PCR, cotton endogenous histone-3 (*AF024716*) was used as an internal standard to normalize the total amount of cDNA in each reaction. Gene-specific primers for the candidate genes were designed with the Beacon Designer software (Table S2). The qRT-PCR experiment was conducted using the TB Green^®^ Premix Ex Taq^TM^ (Tli RNaseH Plus) kit (TaKaRa) according to the manufacturer’s instructions, and all reactions were run as three technical replicates on a QuantStudio 5 Real-Time PCR System. Relative transcript levels were computed using the 2^–ΔCt^ method (ΔCt is the difference of Ct values between the internal standard products and the target gene products).

## 5. Conclusions

RNA-Seq was performed on leaves from drought-treated *G. anomalum* plants to characterize a large number of drought-responsive genes and identify key regulators or pathways involved in drought stress. The analysis yielded 12,322 DEGs related to drought stress; this information could be used in cotton breeding for improving drought tolerance. Ninety GO terms were enriched among the DEGs during drought stress compared to the control (0 d), including 43 in the molecular functions category, 31 in the biological processes group, and 16 in the cellular components cluster, respectively. An enrichment analysis against the KEGG showed the top significantly enriched pathways to be photosynthesis-antenna proteins, amino sugar and nucleotide sugar metabolism, starch and sucrose metabolism, MAPK signaling pathway, glutathione metabolism, and plant hormone signal transduction. In addition, the DEGs included tandemly repeated genes and transcription factors, such as MYB, bHLH, ERF, NAC, WRKY, and bZIP. The data presented here provide deeper insights into the molecular mechanisms of drought stress adaptation in *Gossypium* spp.

## Data Availability

The RNA-seq data are deposited in the BioProject database of NCBI under the accession number of PRJNA697836.

## Supplementary Materials

The following supporting information can be downloaded at: https://www.mdpi.com/article/10.3390/plants12020312/s1, Figure S1. Relative expression level of twelve drought-responsive genes as determined by qRT-PCR and RNA-Seq. The left *y*-axis shows the FPKM value obtained from RNA-Seq data, and the right *y*-axis shows the relative expression obtained from qRT-PCR; Table S1. Summary of RNA-Seq data for *G. anomalum* under drought stress; Table S2: The annotation and expression level of 12,322 differentially expressed genes in *G. anomalum* under drought stress; Table S3: The qRT-PCR primers for twelve drought-responsitive genes; Table S4: GO annotation for differentially expressed genes in *G. anomalum* under drought stress; Table S5: The enrichment GO terms for differentially expressed genes in *G. anomalum* under drought stress; Table S6: The KEGG annotation for differentially expressed genes in *G. anomalum* under drought stress; Table S7: The enrichment KEGG terms for differentially expressed genes in *G. anomalum* under drought stress; Table S8: GO annotation for 776 common differentially expressed genes in *G. anomalum* under drought stress; Table S9: The KEGG annotation for common differentially expressed genes in *G. anomalum* under drought stress; Table S10: The enrichment GO terms for common differentially expressed genes in *G. anomalum* under drought stress; Table S11: The TFs families for differentially expressed genes in *G. anomalum* under drought stress.
