# Supplementary material for: Transcriptome Profiling of Gossypium anomalum Seedlings Reveals Key Regulators and Metabolic Pathways in Response to Drought Stress
Source: Plants (Basel). 2023 Jan 9;12(2):312. doi: 10.3390/plants12020312 (PMC9865944; doi:10.3390/plants12020312)
Supplement: Supplementary file 1 [file plants-12-00312-s001.zip › plants-2009721-Supplemental materials/Supplemental Figures.docx]

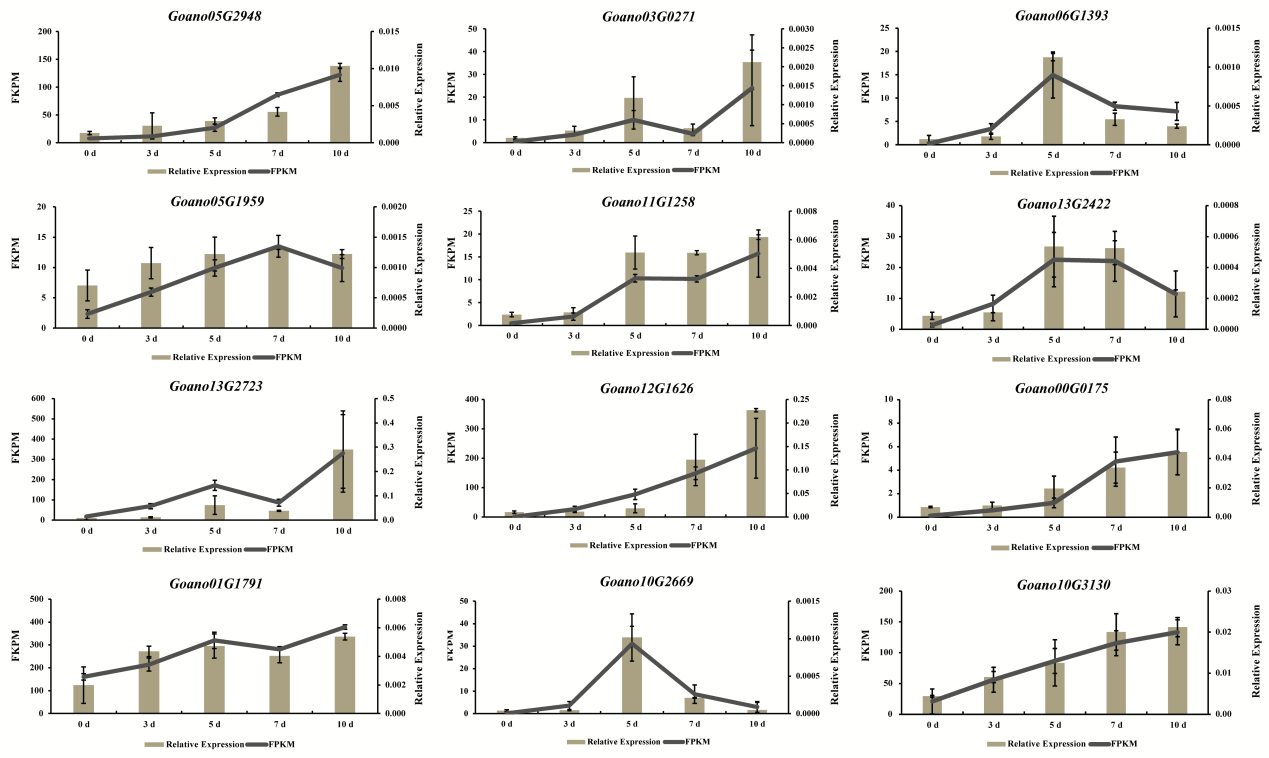


**Figure S1.** Relative expression level of twelve drought-responsive genes as determined by qRT-PCR and RNA-Seq. The left y-axis shows the FPKM value obtained from RNA-Seq data, and the right y-axis shows the relative expression obtained from qRT-PCR.
